# Astrocyte heterogeneity in the neocortex: developmental origins, molecular diversity, and functional implications

**DOI:** 10.3389/fcell.2026.1787840

**Published:** 2026-03-20

**Authors:** Jiafeng Zhou, Riccardo Bocchi

**Affiliations:** Department of Basic Neurosciences, University of Geneva, Geneva, Switzerland

**Keywords:** strocyte development, astrocyte functions, astrocyte lineage, astrocytes heterogeneity, cortical development, single cell RNA sequencing

## Abstract

Astrocytes are increasingly recognized as active regulators of brain development and function rather than passive support cells. Advances in single-cell and spatial transcriptomic technologies have revealed extensive molecular and spatial heterogeneity among astrocytes, much of which emerges early during cortical development and defines distinct subtypes. In the neocortex, this diversity is organized along regional and laminar axes reflecting the interplay between intrinsic developmental programs and local environmental cues. Current evidence supports a model in which neocortical astrocyte diversity arises from lineage-encoded programs that are progressively refined by spatial position and neuronal identity. Astrocytes originate from radial glial cells, undergo local postnatal expansion, and mature into spatially organized domains that constrain diversification. Transcriptomic studies identify region- and layer-associated astrocyte subtypes whose molecular programs correlate with specific functions, including synapse formation, circuit modulation, and behavior. Together, these insights establish astrocyte heterogeneity as a fundamental organizing principle of neocortical circuits and a critical dimension for understanding circuit dysfunction in neurological and neurodevelopmental disorders.

## Introduction

Astrocytes, once regarded as passive support cells, are now recognized as essential organizers of brain development and function. As one of the most abundant cell types in the central nervous system (CNS) (Bass et al., 1971), astrocytes regulate synaptic transmission and plasticity through neurotransmitter uptake and recycling, release gliotransmitters (Allen and Eroglu, 2017; Parpura et al., 1994), and contribute to synapse formation and elimination (Chung et al., 2015; Clarke and Barres, 2013). They also support neurovascular coupling by linking neuronal activity to local blood flow (Iadecola and Nedergaard, 2007) and participate in immune responses, tissue repair, and neuron-glia communication through cytokines, growth factors, and extracellular matrix remodeling (Sofroniew, 2009; Liddelow and Barres, 2017).

Recent advances in single-cell and spatial transcriptomic technologies (Box 1) have transformed our view of astrocytes by revealing extensive molecular and spatial heterogeneity across brain regions (Zhou et al., 2025; Bocchi et al., 2025; Koupourtidou et al., 2024; Ohlig et al., 2021; Bayraktar et al., 2020; Lanjakornsiripan et al., 2018; Batiuk et al., 2020). These studies indicate that astrocytes comprise multiple subtypes with distinct transcriptional identities, spatial distributions, and functional properties. Notably, heterogeneity is already apparent during development (Zhou et al., 2025), suggesting that it is not fully explained by postnatal environmental adaptation alone.

Despite rapid progress, fundamental questions remain unresolved. How are astrocyte molecular programs specified during development? To what extent do lineage-encoded mechanisms versus cell-extrinsic signals, such as neuronal identity and circuit architecture, shape subtype specification and functional specialization? Addressing these questions is particularly important given growing evidence implicating astrocytes in neurological conditions characterized by circuit dysfunction, including neurodevelopmental and neurodegenerative disorders (Parenti et al., 2020; Brandebura et al., 2023; Phatnani and Maniatis, 2015).

In this mini-review, we synthesize recent work on the developmental origins and molecular diversity of neocortical astrocytes, focusing on how cell-intrinsic and cell-extrinsic mechanisms cooperate to generate functional specialization. We discuss emerging evidence linking defined astrocyte subtypes to specific functions and disease-relevant phenotypes and highlight key challenges and future directions for translating astrocyte heterogeneity into mechanistic and therapeutic insights.

Box 1 **Key methodological terms.**

**Spatial transcriptomics:** A class of methods that preserve spatial information while measuring gene expression, enabling the mapping of molecularly defined cell types onto their anatomical and laminar context.

**MERFISH (Multiplexed error-robust fluorescence *in situ* hybridization):** A highly multiplexed spatial transcriptomics method that enables the detection and precise spatial localization of hundreds to thousands of RNA species at single-cell resolution within intact tissue sections.

**scATAC-seq (Single-cell assay for transposase-accessible chromatin sequencing):** A single-cell epigenomic technique that profiles chromatin accessibility across the genome, allowing inference of regulatory elements and transcription factor activity underlying cell-type-specific gene expression programs.

**scRNA-seq (Single-cell RNA sequencing):** A high-throughput approach to quantify gene expression at single-cell resolution, widely used to identify cell types, states, and transcriptional heterogeneity within complex tissues.

**Lineage tracing:** Experimental strategies used to follow the developmental origin and fate of cells over time, often relying on genetic labeling to link progenitor populations to mature cell types.

**Gene ontology (GO) analysis:** A bioinformatic approach that assigns genes to curated functional categories (biological processes, molecular functions, or cellular components), commonly used to identify enrichment of specific pathways or programs within a gene set.

## Generation and maturation of neocortical astrocytes

**FIGURE 1:**
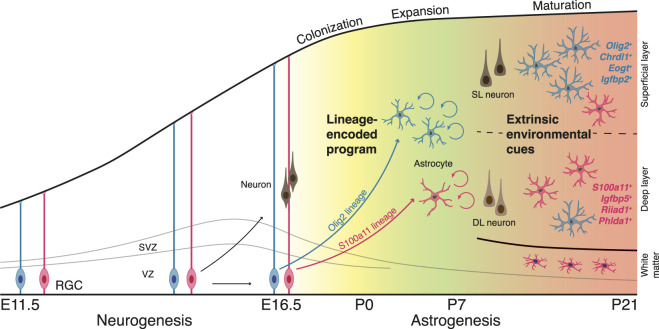
Developmental origins and refinement of neocortical astrocyte identity. Schematic illustrating the temporal and cellular processes that give rise to astrocyte heterogeneity in the developing neocortex. Radial glial cells (RGCs) in the ventricular zone (VZ) initially generate excitatory neurons that populate superficial (SL) and deep (DL) cortical layers during embryonic neurogenesis. A subset of RGCs undergoes a neurogenic-to-gliogenic switch around embryonic day (E)16.5, giving rise to the S100a11 astrocyte lineage, whereas a largely gliogenic Olig2 lineage generates astrocytes with minimal neuronal output. Following birth, immature astrocytes migrate to colonize the cortex and undergo extensive local expansion, propagating lineage-biased identities across the developing cortex. During postnatal maturation, local environmental cues linked to neuronal identity, laminar position, and circuit organization progressively refine astrocyte molecular programs, resulting in region- and layer-associated astrocyte subtypes in the mature neocortex. Together, this sequence highlights how lineage-encoded programs and extrinsic cues act in a coordinated manner to generate stable yet diverse astrocyte identities.

Neocortical astrocytes originate from radial glial cells (RGCs) lining the lateral ventricle in the ventricular zone (VZ), providing the developmental and lineage framework upon which astrocyte diversity is established. In mouse neocortex, RGCs initially generate excitatory neurons from embryonic day (E) 10.5 to E16.5 (Kwan et al., 2012), followed by a neurogenic-to-gliogenic switch that initiates astrocyte production from approximately E16.5 through postnatal stages (until around P7, Figure 1) (Clavreul et al., 2022). This transition is regulated by multiple signaling pathways, including BMP, JAK-STAT (Miller and Gauthier, 2007), Notch and ERK (Gao et al., 2025), and is accompanied by epigenetic remodeling that suppress neurogenic programs while enabling gliogenic gene expression (Williamson et al., 2025).

Newly generated immature astrocytes migrate radially from the VZ toward their final positions within the cortical plate (Clavreul et al., 2022). Although a fraction of astrocytes continues to be generated directly from RGCs during early postnatal stages, most cortical astrocytes arise through local proliferation of immature astrocytes (Ge et al., 2012; Clavreul et al., 2022). This local expansion drives the rapid increase in astrocyte number during the first postnatal week and is a major determinant of astrocyte abundance in the mature cortex (Figure 1) (Williamson et al., 2025; Ge et al., 2012).

Following proliferation, immature astrocytes undergo a progressive maturation process characterized by elaboration and refinement of fine processes (Williamson et al., 2025). During this period, astrocytes acquire their complex, highly ramified morphology (Clavreul et al., 2022) and establish non-overlapping territorial domains, such that each astrocyte occupies a discrete territory with minimal overlap with neighboring astrocytes (Baldwin et al., 2024; Bushong et al., 2004). This maturation process is largely completed by the end of the third postnatal week (Figure 1) (Clavreul et al., 2022).

Importantly, postnatal expansion and territorial organization may provide a developmental framework within which astrocyte diversity emerges. Immature astrocytes are seeded across the neocortex in distinct microenvironments, where they encounter layer- and region-specific extracellular matrix components and cell–cell interactions. These cell-extrinsic cues, together with cell-intrinsic programs inherited from progenitors, shape astrocyte identity. Subsequent local proliferation can then expand these identities into spatially distributed, heterogeneous astrocyte clones across the cortex, setting the stage for the subtype-specific programs discussed below. These developmental steps occur within defined temporal windows (between E16.5 and P21) that coincide with periods of intense synaptogenesis and circuit refinement (Vivi and Di Benedetto, 2024; Milbocker et al., 2021), suggesting that astrocyte diversification is temporally coordinated with neuronal network assembly.

## Molecular diversity of astrocytes

Astrocytes were long considered a relatively homogeneous population, but transcriptomic profiling has revealed extensive molecular diversity across the CNS. These analyses showed that astrocytes acquire distinct molecular identities across brain regions (Batiuk et al., 2020; Herrero-Navarro et al., 2021; Morel et al., 2017; Chai et al., 2017). For instance, astrocytes isolated from neocortex and thalamus display region-specific gene expression programs, including enrichment of *Gbx2* in thalamic astrocytes, highlighting regional specialization of astrocyte identity (Herrero-Navarro et al., 2021). Consistent with this principle, astrocytes in other diencephalic regions also exhibit pronounced molecular heterogeneity and region-specific transcriptional programs, including evidence for Smad4-regulated astrogenesis persisting into adulthood (Ohlig et al., 2021). Regional specialization is further evident when comparing cortical and hippocampal astrocytes, where hippocampal-enriched astrocyte populations express genes such as *Frzb* and *Ascl1*, whereas distinct astrocyte populations characterized by *Unc13c* and *Agt* expression are present in the neocortex but largely absent from the hippocampus (Batiuk et al., 2020). Extending this view, molecular profiling across multiple brain regions identified genes differentially expressed between cortical and subcortical astrocytes, including enrichment of the synapse-regulating gene *Sparc* in hypothalamic and thalamic astrocytes (Morel et al., 2017). Likewise, integrative molecular analyses comparing hippocampal and striatal astrocytes further identified region-specific markers, such as enrichment of μ-crystallin (*Crym*) in striatal astrocytes and higher *Gfap* expression in hippocampal astrocytes (Chai et al., 2017). Together, these studies established regional identity as a major axis of astrocyte molecular diversity.

More recent studies have revealed intra-regional astrocyte diversity, uncovering additional layers of molecular heterogeneity linked to spatial organization. In the neocortex, astrocytes display pronounced layer-associated transcriptional differences. Single-cell and spatial transcriptomic analyses revealed that astrocytes residing in superficial versus deep cortical layers express distinct gene sets. For example, *Chrdl1* is preferentially expressed in astrocytes located in upper cortical layers, whereas *Il33* and *Id3* are enriched in deep-layer astrocytes (Lanjakornsiripan et al., 2018; Bayraktar et al., 2020). Additional layer-restricted markers include *Scel*, selectively expressed in layer 4 astrocytes, and *Id1*, enriched in astrocytes of layers 5-6 (Bayraktar et al., 2020). These molecular gradients closely mirror cortical lamination and suggest that astrocyte identity is aligned with local neuronal architecture (Figure 1).

**FIGURE 2:**
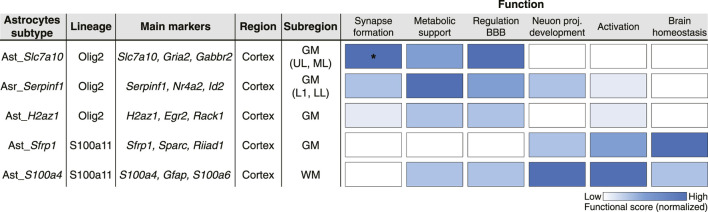
Integrative summary of molecular identity, developmental lineage, spatial distribution, and functional specialization of cortical astrocyte subtypes. Molecularly defined astrocyte subtypes in the mouse cortex are organized by developmental lineage (Olig2 vs. S100a11), key marker genes, regional and subregional localization, and enrichment of functional gene programs. Functional scores, derived from previously published transcriptomic analyses (Zhou et al., 2025), were computed as expression-based scores for curated gene sets associated with six main astrocyte functional categories (synapse formation, metabolic support, regulation of BBB, neuron projection development, activation, and brain homeostasis). Gene sets were derived from gene ontology (GO)-based annotations (for complete list see Supplementary Data 8 of (Zhou et al., 2025)), and subtype-specific scores were calculated as the average log-normalized expression of genes within each set using Seurat’s CellCycleScoring() function. Scores are normalized within each functional category and indicate relative enrichment across subtypes. This integrative view illustrates how astrocyte subtypes occupy distinct positions within a multidimensional functional space shaped by developmental origin and spatial context rather than representing uniform functional classes. GM, grey matter; WM, white matter; UL, upper layers; ML, middle layers; LL, lower layers; L1, layer 1; BBB, blood–brain barrier; proj., projections; * indicates functionally validated roles (Zhou et al., 2025).

More comprehensive single-cell analyses spanning all cortical layers and white matter have further refined the molecular organization of neocortical astrocytes, revealing multiple molecularly distinct subtypes with defined spatial distributions (Zhou et al., 2025). In addition to populations enriched in layer 1 and white matter, gray matter astrocytes segregate into subtypes preferentially localized to upper, middle, and lower cortical layers (Figure 2). The convergence of these findings with independent datasets (Lanjakornsiripan et al., 2018; Bayraktar et al., 2020) supports a model in which neocortical gray matter astrocytes are broadly organized into three laminar classes aligned with cortical depth (upper, middle and lower layers). White matter astrocytes also exhibit pronounced molecular heterogeneity, with two distinct subtypes showing divergent transcriptional profiles (Bocchi et al., 2025). Notably, one subtype is characterized by high expression of progenitor-associated genes (*e.g., Sox4*, *Ascl1*, and *Hmgb2*) and enrichment of cell cycle- and proliferation-related programs, indicating a proliferative state that was further validated by *in vivo* proliferation assays (Bocchi et al., 2025).

Collectively, these studies establish molecular diversity as a fundamental property of astrocytes, structured along both regional and intra-regional axes. Importantly, the enrichment of synapse-related genes, signaling molecules, and extracellular matrix components within specific astrocyte subtypes provides a molecular framework for functional specialization. How these molecular programs arise during development and how they translate into subtype-specific astrocyte functions are key questions addressed in the following sections.

## Cell-intrinsic and cell-extrinsic mechanisms shaping astrocyte diversity

Astrocyte diversity arises through the combined action of cell-intrinsic programs and cell-extrinsic environmental cues (Clavreul et al., 2019; Bayraktar et al., 2020; Lanjakornsiripan et al., 2018). Rather than acting as opposing mechanisms, lineage-encoded programs and local signals operate sequentially and cooperatively to refine astrocyte molecular identity during development (Figure 1). Early lineage-tracing studies using combinatorial labeling approaches, in which individual astrocyte progenitors are marked with distinct colors to follow their progeny, demonstrated that individual astrocyte progenitors can generate progeny with diverse morphologies and spatial positions, indicating that astrocyte identity retains a degree of plasticity and can be shaped by local context (Clavreul et al., 2019). Consistent with a prominent role for extrinsic cues, perturbation of neuronal lamination profoundly alters astrocyte layer-specific gene expression (Bayraktar et al., 2020; Lanjakornsiripan et al., 2018). In mouse models with disrupted cortical layering, including *Satb2*, *Dab1*, and Reeler mutants, astrocytes fail to establish normal laminar molecular identities: genes typically enriched in superficial-layer astrocytes, such as *Chrdl1* and *Eogt*, are reduced or ectopically expressed, whereas markers associated with deep-layer astrocytes, including *Il33* and *Id3*, show inverted or diffuse expression patterns (Bayraktar et al., 2020; Lanjakornsiripan et al., 2018). These findings indicate that neuronal organization and circuit architecture provide instructive environmental cues that shape astrocyte molecular identity.

Alongside these extrinsic influences, multiple lines of evidence support a critical contribution of cell-intrinsic, lineage-encoded mechanisms to astrocyte diversity. Lineage-tracing studies have shown that progenitors in the dorsal, intermediate, and ventral forebrain give rise to astrocytes that remain spatially confined to their respective regions, suggesting that astrocyte identity is specified, at least in part, by regionally restricted progenitor populations (Tsai et al., 2012). A similar principle operates in the spinal cord, where astrocytes derived from distinct progenitor domains occupy discrete territories defined by their embryonic origins (Tsai et al., 2012). Within the neocortex, clonal analyses of progenitors labeled at E14 revealed that individual clones preferentially comprise either pial or protoplasmic astrocytes, supporting the existence of progenitors intrinsically committed to generate specific astrocyte subtypes (Garcia-Marques and Lopez-Mascaraque, 2013).

The integration of lineage tracing (Box 1) with single-cell transcriptomics has further refined this view, revealing the coexistence of multipotent and fate-biased astrocyte lineages. In the mouse hippocampal neuroepithelium, fate-biased lineages have been identified in which one lineage primarily generates neurons and gray matter astrocytes, whereas another lineage predominantly produces glial cells, including oligodendrocyte and white matter astrocytes (Ratz et al., 2022). Similar lineage organization has been reported in the developing mouse neocortex, where both multipotent and fate-restricted lineages coexist (Zhou et al., 2025). One lineage derives from Emx1-positive progenitors that initially generate neurons before switching to astrocyte production (S100a11 lineage), whereas a second lineage with minimal neuronal output, predominantly produces a distinct subset of astrocytes marked by Olig2 (Olig2 lineage, Figure 1) (Zhou et al., 2025). Thus, lineage identity contributes substantially to the molecular, spatial, and morphological diversity of cortical astrocytes.

Together, these studies support a model in which astrocyte diversity emerges from the interplay between lineage-encoded programs and local environmental cues. The relative contribution of these mechanisms likely varies across brain regions and developmental stages, providing both robustness and flexibility to astrocyte subtype specification.

## Functional diversity of astrocytes

The molecular heterogeneity of astrocytes suggests that distinct astrocyte subtypes perform specialized functions (Batiuk et al., 2020; Zhou et al., 2025; Bayraktar et al., 2020). Although direct functional evidence for astrocyte subtype specialization is still emerging, recent cortical studies together with work in other brain regions have begun to move beyond correlative associations by providing proof-of-principle that specific astrocyte populations exert defined circuit-level and behavioral effects (Ollivier et al., 2024; de Ceglia et al., 2023; Zhou et al., 2025).

Compelling evidence for astrocyte functional specialization comes from the identification of astrocyte subpopulations capable of gliotransmission (de Ceglia et al., 2023). A discrete population of hippocampal astrocytes expressing synaptic-like glutamate-release machinery, including *Slc17a7* (Vglut1), was shown to mediate glutamatergic signaling at defined circuit sites (de Ceglia et al., 2023). Astrocyte-specific disruption of glutamate release from these cells impaired synaptic plasticity and memory in cortico-hippocampal circuits and altered dopamine levels in the nigrostriatal pathway, exemplify a direct role for a molecularly distinct astrocyte subtype in regulating long-range circuit function and behavior.

Astrocyte functional heterogeneity is also evident in the striatum, where spatially restricted astrocyte populations regulate local circuit dynamics (Ollivier et al., 2024). In this region, astrocytes expressing μ-crystallin (*Crym*) are selectively enriched in the dorsomedial and centromedial striatum. Loss of *Crym* disrupted astrocyte-mediated control of presynaptic GABA release via GABA transporter 3 (GAT3), leading to altered excitation–inhibition balance in medium spiny neurons and the emergence of perseverative behaviors.

In the neocortex astrocytes play critical roles in synapse formation and maturation through synaptogenic and anti-synaptogenic factors, including glypicans 6 (*Gpc6*) (Williamson, Murali, and Deneen, 2025), chordin-like 1 (*Chrdl1*) (Blanco-Suarez et al., 2018) and members of the protocadherin (*Pcdh*) gene family (Yasuda et al., 2007; Hoshina et al., 2013). Importantly, these factors are not uniformly expressed across all astrocytes but instead segregate among molecularly and developmentally distinct subtypes (Zhou et al., 2025). Genes promoting synapse formation, such as *Gpc6*, *Sparcl1* (Kucukdereli et al., 2011), *Chrdl1* (Blanco-Suarez et al., 2018) and *Pcdh17* (Hoshina et al., 2013) are enriched in the S100a11 lineage, whereas genes that limit synaptogenesis, including *Sparc* (Kucukdereli et al., 2011) and *Pcdh8* (Yasuda et al., 2007), are preferentially expressed in the Olig2 lineage. Astrocytes of the latter lineage localize near excitatory synapses (Tatsumi et al., 2021; Tatsumi et al., 2018), and lineage-specific perturbation of their development results in reduced dendritic spine density and synapse abundance (Zhou et al., 2025), providing direct evidence that astrocyte subtype identity shapes synaptic architecture *in vivo* (Figure 2).

Together, these studies establish a causal link between molecular heterogeneity and functional specialization, demonstrating that astrocyte subtypes act as active and specialized regulators of neural circuit development and function rather than interchangeable support cells.

## Perspectives and disease implications

Understanding astrocyte heterogeneity is essential for deciphering how these cells fulfil their diverse roles across the CNS. Astrocytes regulate synaptic transmission and plasticity by controlling neurotransmitter uptake, recycling, and release, secrete gliotransmitters, and modulate synapse formation and elimination (Allen and Eroglu, 2017; Parpura et al., 1994; Chung et al., 2015; Clarke and Barres, 2013). Their unique positioning at the interface between neurons and blood vessels enables them to coordinate neurovascular coupling and metabolic support (Iadecola and Nedergaard, 2007). The emerging view that these functions are differentially distributed across molecularly and developmentally distinct astrocyte subtypes provides a new framework for understanding circuit development and homeostasis. A central unresolved question is how developmental origin and lineage are translated into functional specialization of astrocytes. Despite major advances in defining astrocyte molecular diversity, the causal links between lineage encoded programs, subtype-specific molecular profiles, and functional outputs remains incompletely understood.

**FIGURE 3:**
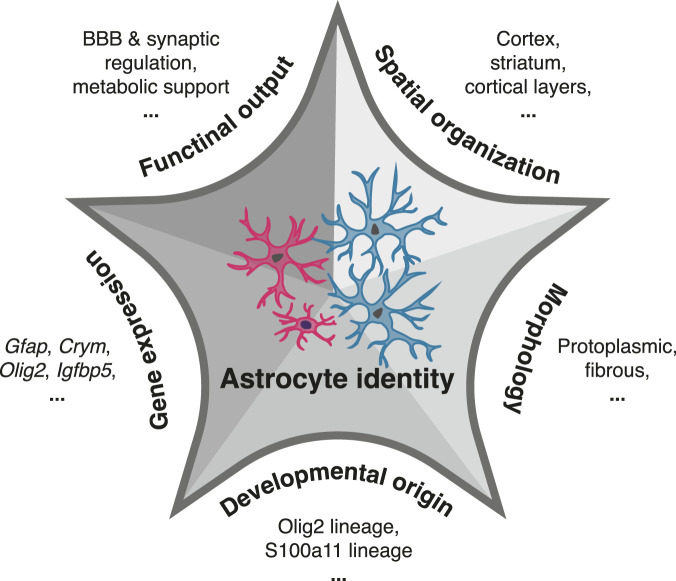
Conceptual framework for astrocyte identity. Astrocyte heterogeneity is defined by the convergence of multiple organizational dimensions, including lineage-dependent developmental programs, subtype-specific molecular signatures, morphological specialization, spatial positioning within brain regions and cortical layers, and distinct functional roles. Their integration defines stable yet adaptable astrocyte identities.

Addressing this challenge requires moving beyond transcriptomic profiles alone, which are insufficient to define biologically meaningful astrocyte subtypes. Robust subtype definitions will require multimodal integration of gene expression with morphology, spatial organization, developmental origin and functional output, analogous to the criteria used to define neuronal cell types (Figure 3). Integrating these complementary dimensions will be essential to distinguish stable astrocyte identities from dynamic or context-dependent cellular states and to establish a reproducible, mechanistically informative taxonomy of astrocyte diversity. Future studies that integrate single-cell transcriptomics (e.g., scRNA-seq, Box 1) with epigenomic profiling (e.g., scATAC-seq, Box 1), spatial transcriptomics (e.g., MERFISH, Box 1), and lineage-resolved genetic perturbations will be essential for linking astrocyte molecular programs to specific functions. Such approaches will also help clarify how interactions between astrocytes and neighboring neurons, vasculature, and immune cells contribute to the establishment, maintenance, and plasticity of astrocyte subtype identity across development and adulthood.

Accounting for astrocyte heterogeneity has important implications for disease. A growing body of evidence implicates astrocytes in neurological conditions, including neurodevelopmental disorders, neurodegenerative diseases, and brain injury (Cabezas et al., 2014; Sauer et al., 2021; Burda et al., 2016). Although astrocyte involvement in these conditions is increasingly well established, the specific contribution of astrocyte molecular and functional heterogeneity to disease pathogenesis remains poorly understood. In ischemia, trauma, and epilepsy, blood–brain barrier (BBB) disruption and metabolic stress are central pathological features. Astrocytes are key regulators of vascular integrity, ion and neurotransmitter homeostasis, and metabolic coupling to neurons. Thus, astrocyte subtypes enriched for BBB regulation or metabolic support gene programs may be preferentially engaged or become maladaptive under these conditions. While phenotypic remodeling of perivascular astrocytes has been documented, direct causal evidence linking defined molecular subtypes to BBB breakdown or metabolic failure remains limited (Cabezas et al., 2014). In neurodevelopmental disorders, including autism spectrum disorder, altered synaptic connectivity and circuit organization represent core features. Astrocyte subtypes enriched for synaptogenic or circuit-modulatory gene programs could, in principle, influence disease risk by altering the balance of synapse formation, maturation, or elimination during critical developmental windows (Sauer et al., 2021). At present, these links remain largely hypothesis-driven, although emerging transcriptomic and functional data support the plausibility of subtype-specific contributions (Zhou et al., 2025). Taken together, astrocyte involvement in disease is unlikely to be uniform across the population. Rather, disease susceptibility and progression may reflect the selective engagement, intrinsic vulnerability, or maladaptive state transitions of specific astrocyte subtypes whose functional specialization aligns with the dominant pathological features of a given disorder. Defining the molecular and cellular mechanisms that connect astrocyte subtype identity to disease initiation and progression remains an important challenge for the field.

A major challenge for the field is therefore to move beyond bulk manipulations of astrocytes toward subtype- and lineage-specific approaches. Defining how selective perturbations of distinct astrocyte populations influence synaptic development, circuit maturation, metabolic support, blood-brain barrier regulation and other astrocyte functions will be essential for establishing causal links between astrocyte diversity and brain disorders. Ultimately, integrating developmental, molecular, and functional perspectives on astrocyte heterogeneity (Figure 3) will refine our understanding of neocortical organization and may enable more precise strategies to modulate pathological astrocyte states while preserving essential homeostatic functions.
